# Investigation of the In Vivo, In Vitro, and In Silico Wound Healing Potential of *Pinctada martensii* Purified Peptides

**DOI:** 10.3390/md20070417

**Published:** 2022-06-26

**Authors:** Ting Zhang, Faming Yang, Xiaoming Qin, Xianmei Yang, Chaohua Zhang, Zhaoyi Wan, Haisheng Lin

**Affiliations:** 1 College of Food Science and Technology, Guangdong Ocean University, Zhanjiang 524088, China; zhting95@163.com (T.Z.); yangfm0123@163.com (F.Y.); 13414884976@163.com (X.Y.); zhangch2@139.com (C.Z.); spring5water@163.com (Z.W.); haishenglin@163.com (H.L.); 2 Marine College, Shandong University, Weihai 264209, China; 3 Guangdong Provincial Key Laboratory of Aquatic Product Processing and Safety, Guangdong Ocean University, Zhanjiang 524088, China; 4 National Research and Development Branch Center for Shellfish Processing (Zhanjiang), Zhanjiang 524088, China; 5 Guangdong Province Engineering Laboratory for Marine Biological Products, Guangdong Ocean University, Zhanjiang 524088, China; 6 Guangdong Provincial Engineering Technology Research Center of Marine Food, Guangdong Ocean University, Zhanjiang 524088, China; 7 Collaborative Innovation Center of Seafood Deep Processing, Dalian Polytechnic University, Dalian 116034, China

**Keywords:** *Pinctada martensii* purified peptides, molecular docking, traceless healing

## Abstract

Previous studies found that both oral and topical administration of enzymatic digestion products < 3 K Da ultrafiltration fractions of *Pinctada martensii* mantle (PMPs) had pro-healing effects. Thus, we further purified them by Sephadex-G25 and screened them by cellular assays to obtain *Pinctada martensii* purified peptides (PMPPs). In this study, we explored the mechanism of PMPPs on wound healing by in vivo, in vitro, and in silico experiments. LC-MS/MS results showed that PMPPs consisted of 33 peptides with molecular weights ranging from 758.43 to 2014.04 Da, and the characteristic peptide was Leu-Asp. The results of cellular assays showed that PMPPs promoted the proliferation of human skin fibroblasts (HSF) (135%) and human immortalized keratinocyte (HaCaT) cells (125%) very significantly at 12.5 μg/mL. The in vivo results showed that PMPPs could achieve scarless healing by inhibiting the inflammatory response, accelerating the epithelialization process, and regulating collagen I/III ratio. The optimal peptide sequence FAFQAEIAQLMS of PMPPs was screened for key protein receptors in wound healing (EGFR1, FGFR1, and MMP-1) with the help of molecular docking technique, which also showed to be the key pro-healing active peptide sequence. Therefore, it may provide a therapeutic strategy with great potential for wound healing.

## 1. Introduction

Wound repair involves three cross-linking phases: inflammatory response, cell proliferation, and tissue reconstruction [1]. After the skin is traumatized, an inflammatory reaction period begins, which lasts 3–5 days. Neutrophils accumulate at the wound site, cleaning up tissue debris and bacteria [2], which can lead to cell migration, proliferation and differentiation, and form granulation tissue, as well as promote the regeneration of collagen products and endothelial cells [3]. Finally, in the post-traumatic tissue remodeling period, a large amount of extracellular matrix (ECM) and type III collagen is gradually degraded, and type I collagen is meanwhile generated [4]. However, if wounds are not treated in time, it can cause wound infection, pain, the formation of thickened scars, and slow wound healing to affect esthetics and quality of life [5]. Therefore, accelerating skin wound closure and suppressing scarring are essential for patients with tissue defects.

Marine active substances have unique functions due to marine biodiversity and extremely complicated living environments [6]. Hence, these substances have great potential of use in the fields of food, medicine, health care, and cosmetics [7,8,9]. Presently, marine active substances are extracted from marine organisms such as fish, shrimp, sponges, seaweed, fungi, and so on [10] to develop marine drugs [11]. For example, oral salmon collagen peptides promote skin wound healing and angiogenesis in mice [12,13]. Oral low-molecular-weight peptides from *Theragra chalcogramma* can promote skin wound healing [14]. In addition, our previous study showed that oral and topical active peptides of *Pinctada martensii* could accelerate skin wound healing [15,16]. However, there is still a lack of reports of natural peptide drugs and products with clear structures that promote healing, which is hampered by the complex process of drug discovery. Excitingly, application of molecular docking technology can efficiently screen drug candidates, and save time and cost [17].

Noteworthy, purified peptides derived from *Pinctada martensii* or even marine shellfish to promote healing had received less reports and attention. In this study, we further separated and purified the obtained ultrafiltration fractions through gel chromatography and verified the activity using cellular experiments. The peptides fractions were identified by LC-MS/MS, and animal experiments were selected to investigate their healing-promoting mechanisms. Finally, the optimal peptide sequences were screened by molecular docking and their molecular mechanisms were analyzed. The aim is to provide new drugs and new ideas for wound healing treatment.

## 2. Results

### 2.1. Screening and Purification of Peptides Fractions

**Figure 1:**
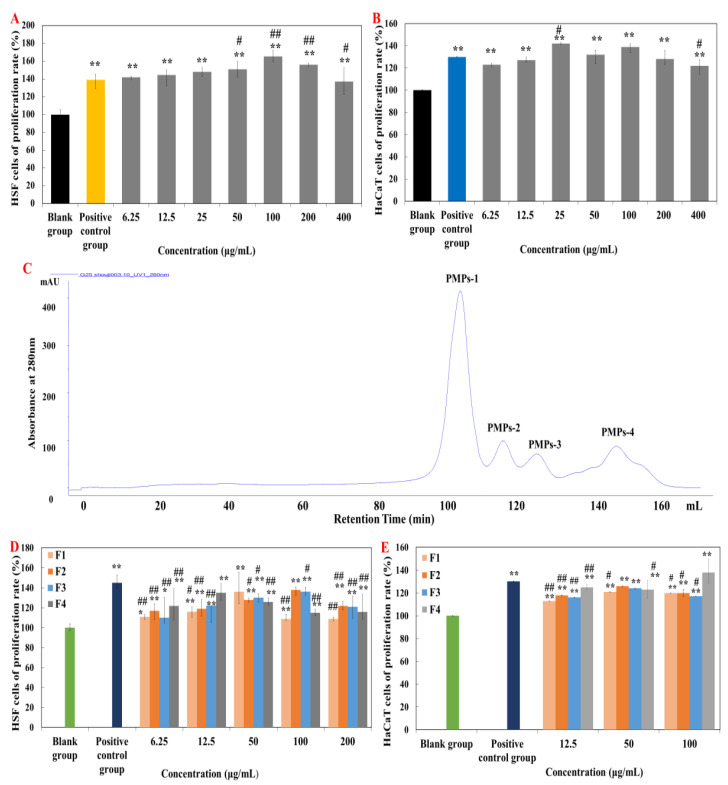
Effects of purified peptides on cells proliferation. (**A**,**B**) Effects of PMPs on cells proliferation. (**C**) Gel permeation chromatogram of PMPs on a Sephadex G-25 column. (**D**,**E**) Effects of PMPPs on cells proliferation. Note: “*” means significantly different compared with control group (*p* < 0.05), “**” means highly significant difference compared with control group (*p* < 0.01), “#” means significantly different compared with positive control group (*p* < 0.05), “##” means highly significant difference compared with positive control group (*p* < 0.01). The concentration of the positive control group is 10 ng·mL^−^^1^.

In vitro cellular experiments are an effective means of activity verification and fraction screening [15,16]. Firstly, effect of the obtained PMPs on HSF and HaCaT cells proliferation were explored (Figure 1A,B). Figure 1A showed that the positive control group (Human FGF-basic) and each concentration of PMPs significantly promoted the proliferation of HSF cells (*p* < 0.01). At the concentration of 100 μg·mL^−^^1^, the proliferation rate of HSF cells (165.31%) was the highest and significantly higher than that of the positive control group (*p* < 0.01). Simultaneously, at the concentration of 25 μg·mL^−1^, the proliferation rate of HaCaT cells (142%) was significantly different (*p* < 0.05) and significantly higher than that of the positive control group (rhEGF) compared with the blank control group (*p* < 0.01) (Figure 1B).

Therefore, Sephadex G-25 gel chromatography was selected for separation and purification of PMPs (Figure 1C). Four components were respectively collected and named as PMPs-1, PMPs-2, PMPs-3, and PMPs-4 (protein contents were 3.087 mg·mL^−1^, 3.395 mg·mL^−1^, 3.494 mg·mL^−1^, and 3.912 mg·mL^−1^). Samples were lyophilized and reserved for cell experiments.

Figure 1D showed that the positive control group (Human FGF-basic) significantly promoted the proliferation of HSF cells (*p* < 0.01), and the proliferation rate was higher than in other groups. In addition, all groups of PMPs also significantly promoted the proliferation of HSF cells, with the PMPs-4 component being the most effective in promoting the proliferation of HSF cells.

Figure 1E showed the positive control group (rhEGF) (administration concentration of 10 μg·mL^−^^1^, the proliferation rate of 130%) significantly promoted the proliferation of HaCaT cells (*p* < 0.01). The proliferation rate of PMPs-1, PMPs-2, and PMPs-3 components were the highest at the concentration of 50 μg·mL^−^^1^. However, when the concentration of PMPs-4 was 12.5 μg·mL^–1^, the increment rate was 125%, which was higher than that of F1, F2, and F3. So, the PMPs-4 component has the best effect in promoting the proliferation of HaCaT cells. Accordingly, PMPs-4 component as *Pinctada martensii* purified peptides (PMPPs) was selected for subsequent studies in this study.

### 2.2. Identification of PMPPs Peptide Sequences by LC-MS/MS

**Figure 2:**
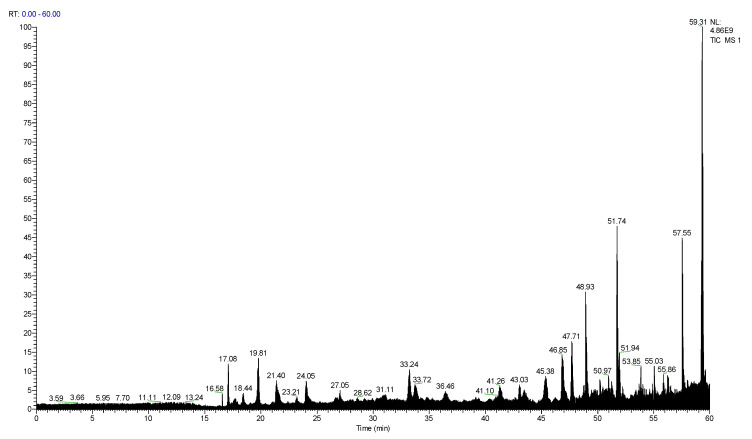
Total ion chromatogram of PMPPs.

By primary mass spectrometry, it was found that PMPPs of 33 peptides had molecular weight ranging from 758.43 to 2014.04 Da (Figure 2).

Peptide fingerprinting of 20 characteristic peptides in the PMPPs was analyzed using an LC-MS/MS. The molecular weight of PMPPs was in the range 947.43–1992.06 Da (amino acid residue 7–17) (Table 1). One peptide fragment of Leu-Asp recurred in the 20 characteristic peptide sequences of PMPPs; Phe, Lys, and Arg recurred at the beginning of the characteristic peptide; and Glu reappeared at the tail of the characteristic peptide. Additionally, Leu, Ser, and Lys were found in the middle of PMPPs characteristic peptides.

### 2.3. Effect of PMPPs on Wound Healing in Mice

#### 2.3.1. Macroscopic Effects of PMPPs on Wound Healing

**Figure 3:**
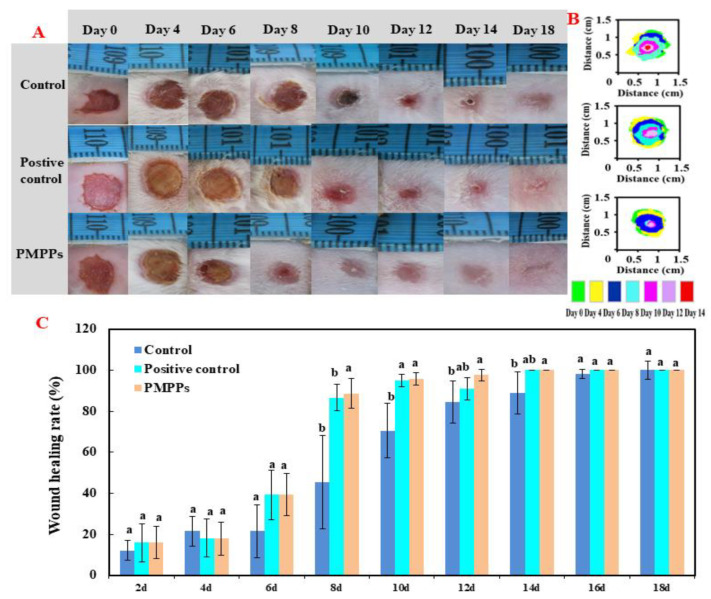
Effect of PMPPs on skin wound healing in mice. (**A**) Photographs of representative wounds on mice on days 0, 4, 6, 8, 10, 12, 14, and 18. (**B**) The area traces of wound healing in each group on days 0, 4, 6, 8, 10, 12, 14, and 18. (**C**) Wound healing rate of each group (calculated every two days). Values are expressed as mean ± SD, *n* = 6. Note: The same superscript letters indicate no significant difference (*p* > 0.05), and different superscript letters indicate significant differences (*p* < 0.05). The drug of positive control group was Jing Wanhong ointment.

Figure 3A,B visualized the healing process of each group of wounds, in which the topical administration of PMPPs significantly accelerated the wound healing. Figure 3C showed that there was no significant difference in the wound healing rate among the groups 4–6 days after modeling (*p* > 0.05). On days 8–14, topical administration of PMPPs significantly promoted the epithelialization process of the wounds compared with the control group and completed wound healing on day 14 (*p* < 0.05). However, wound healing was achieved in the control group on day 18, also demonstrating the advantages of topically administered PMPPs.

#### 2.3.2. Effects of the PMPPs on Wound Cytokines in Mice

**Figure 4:**
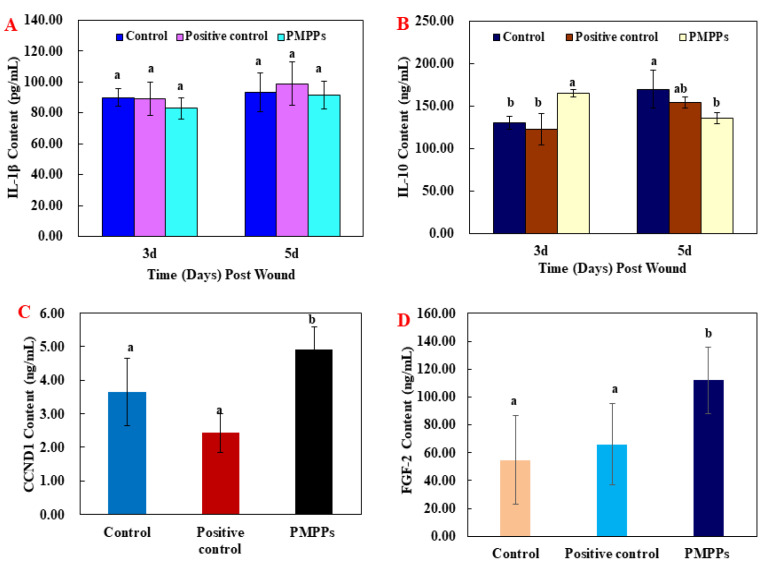
The effect of the PMPPs on wound cytokines in mice. (**A**) IL-1β content of mice in each experimental group on the 3rd and 5th day after animal model establishment. (**B**) IL-10 content of mice in each experimental group on the 3rd and 5th day after animal model establishment. (**C**) CCND1 content of mice in each experimental group on the 7th day after animal model establishment. (**D**) FGF-2 content of mice in each experimental group on the 7th day after animal model establishment. Note: the same superscript letters indicate no significant difference (*p* > 0.05).

The results of the inflammatory factor assay in Figure 4A,B showed that the topical administration of PMPPs at day 3 achieved an anti-inflammatory effect by inhibiting the secretion of the pro-inflammatory factor IL-1β (*p* > 0.05) and significantly promoting the secretion of the anti-inflammatory factor IL-10 (*p* < 0.05), while matching and decreasing the trend at day 5.

Moreover, the results of growth factors assay showed that topical administration of PMPPs significantly promoted the secretion of CCND1 and FGF-2 compared with the two control groups (*p* < 0.05) (Figure 4C,D).

#### 2.3.3. Effects of PMPPs on Wound Tissue Regeneration

**Figure 5:**
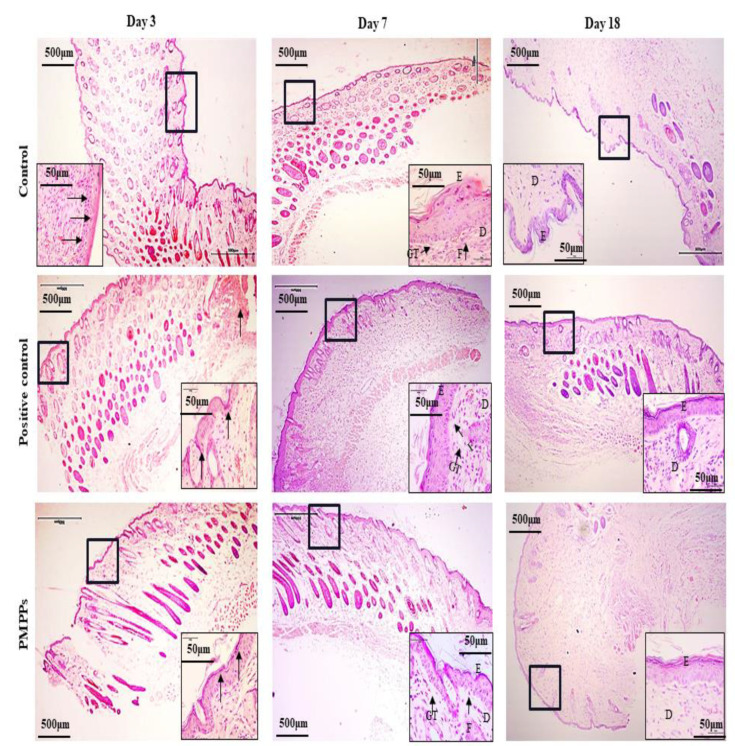
H&E stain histological analysis (4×). Note: Black thick arrows indicate inflammatory cell infiltration. Letters D, E, F, and GT represent the dermis layer, the epidermal layer, fibroblasts, and granulation tissue, respectively.

Figure 5 and Figure S1 demonstrate the effect of topical administration of PMPPs on skin wound healing in mice by H&E staining microscopy results. On day 3, inflammatory cells from the two control groups infiltrated the wounds. However, the inflammatory response was weaker in the PMPPs group, which is consistent with the results in Figure 4A,B. At 7th days, the epidermal layer of the wounds in the negative control group was not completely healed, and collagen fibers in the dermis were sparse and few. In the positive control and PMPPs groups, the wounds formed a coherent epidermis and more granulation tissue in the dermis. Eighteen days after mock-up, the epidermis and dermis of the positive control and PMPPs groups were repaired similarly to normal skin compared to the negative control group, which is consistent with the results in Figure 3A.

**Figure 6:**
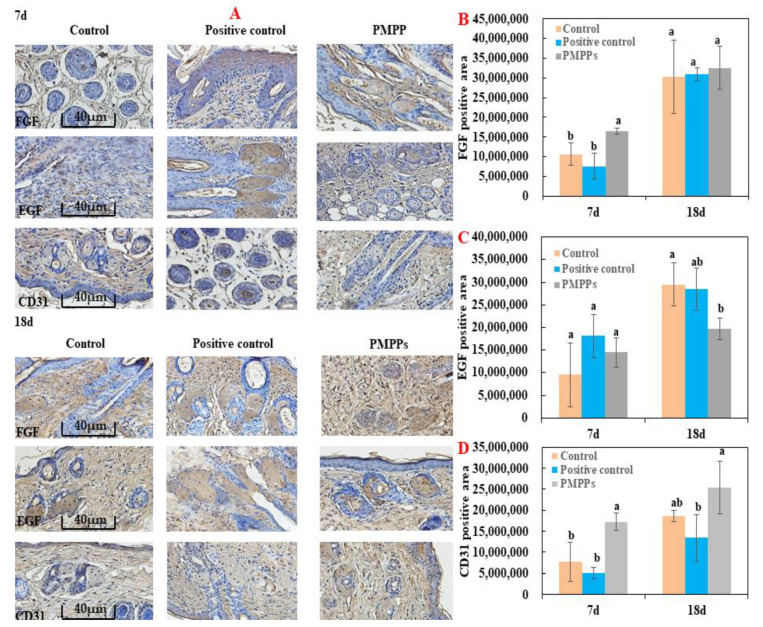
Immunohistochemical analysis of the marine bioactive peptide PMPPs on skin wounds in mice. (**A**) Representative images of FGF, EGF, and CD31 immunostaining of wounds in each group on days 7 and 18. (**B**) On the 7th and 18th day, the expression of FGF in wounds of each group after trauma. (**C**) EGF expression in wounds of each group after 7 days and 18 days of trauma. (**D**) CD31 expression in wounds of each group after 7 days and 18 days of trauma. Note: the same superscript letters indicate no significant difference (*p* > 0.05).

As shown in Figure 6, immunohistochemistry was chosen to evaluate fibroblasts (FGF), epidermopoietic cells (EGF), and vascular regeneration (CD31) during trauma repair. Topical administration of PMPPs significantly promoted the expression of FGF and CD31 at day 7 compared with the control and positive control groups (*p* < 0.05), which is consistent with the results in Figure 4C,D. However, at day 18, the groups gradually converged, which is consistent with the results in Figure 3 and Figure 5. However, there was no significant difference in the expression of EGF in the PMPPs group at day 7 compared to the other groups (*p* > 0.05). This suggests that topical administration of PMPPs accelerates the process of wound epithelialization by promoting fibroblast proliferation and vascular regeneration.

### 2.4. Effects of PMPPs on Wound Collagen and Scar Formation

The scar reduction rate, the image of Sirius red staining, and the ratio of type I/III collagen were analyzed as the important basis for the degree of scar reduction in each experimental group. Under a polarized light microscope, type I fibers are tightly packed, show strong birefringence, and appear as yellow or red fibers. Type III fibers show weak birefringence and appear green.

**Figure 7:**
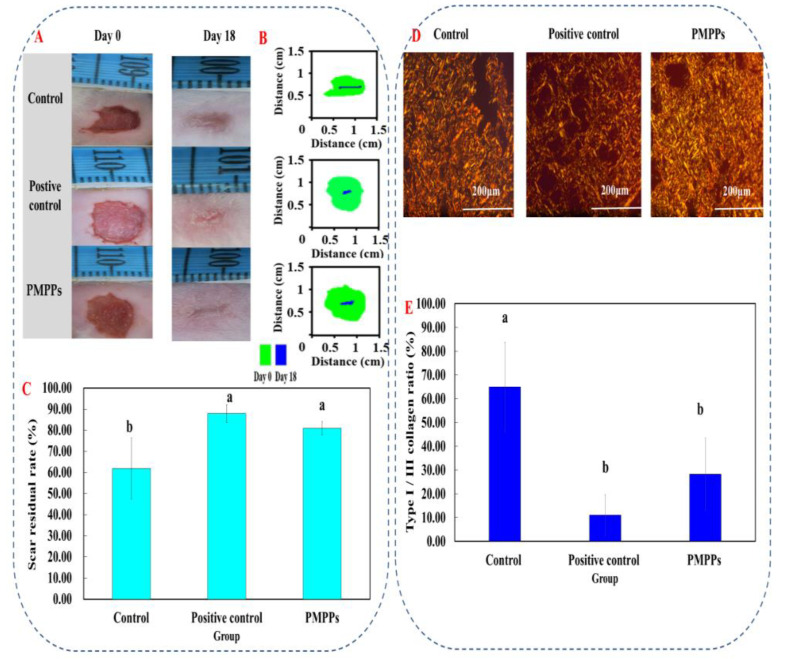
Effects of topical administration of PMPPs on wound collagen and scar formation. (**A**) Photographs of representative wounds in mice on days 0 and 18. (**B**) The area traces of scar reduction in each group on days 0, 4, 6, 8, 10, 12, 14, and 18. (**C**) The rate of scar reduction in each group. (**D**) Representative Sirius red-stained photos in collagen synthesis (magnification: ×200). Collagen III was green and collagen I was yellowish red. (**E**) The expression of collagen I/III after 18 days of modeling. Note: Different superscript letters on the same day indicate significant (*p* < 0.05) and non-significant (*p* > 0.05) differences between groups, respectively.

Firstly, Figure 7A,B visualize the effect of each group on the scar residue on the wounds; compared with the control group, all the administered groups effectively inhibited the scar residue, which is consistent with the results in Figure 7C. Then, Figure 7D showed the microscopic results denoting that compared with the two control groups, the I/III collagen in the PMPPs group was uniform in composition, and the collagen was knitted orderly and densely on day 18. Figure 7E showed that the I/III collagen ratio in the PMPPs group was significantly lower than that in the negative control group on the 18th day (*p* < 0.05).

By measuring the content of TGF-β1, TβRII, and Smad 7 (It was an important inhibitory regulatory protein), the effect of PMPPs on the TGF-β/Smad signaling pathway was studied. ELISA results revealed that the protein expression levels of TGF-β and TβRII in the PMPPs group were significantly higher than those in the negative control group (*p* < 0.05) (Figure S2). Figure S2C showed that the Smad 7 content of the PMPPs group was significantly lower than that of the positive control group (*p* < 0.05). Although there was no significant difference in the content of Smad 7 among the groups, the PMPPs group had the lowest content of Smad 7 (*p* > 0.05). This indicated that topical administration of PMPPs could promote collagen secretion through the TGF-β/Smad signaling pathway.

### 2.5. Molecular Docking

Based on the results in Table 1, we selected a total of eight peptide sequences with scores ≥ 40 (the higher the peptide sequence score in the mass spectrometry results, the higher the reliability) in PMPPs and docked them with wound healing-related protein receptors EGFR1, FGFR1, and MMP-1 for the purpose of screening key peptide sequences and further elucidating the healing-promoting mechanism of PMPPs.

**Figure 8:**
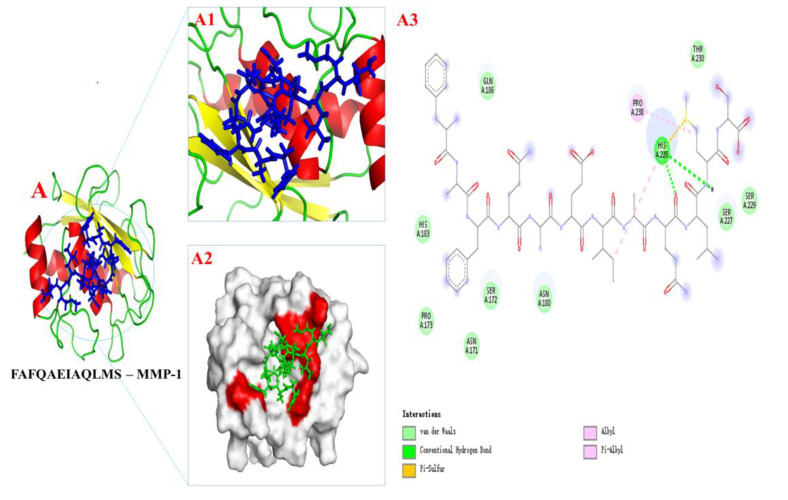
The docking results of PMPPs (FAFQAEIAQLMS) with protein receptors MPP-1. (**A1,A2**) 3D structure of PMPPs—MMP-1 and the process of binding interaction between them. (**A3**) 2D interaction diagram of PMPPs—MMP-1.

The optimal binding model of PMPPs (FAFQAEIAQLMS)—MMP-1 is shown in Figure 8A, with a minimum binding energy of 5.12 kcal/mol, indicating that a stable complex was formed between PMPPs (FAFQAEIAQLMS) and MMP-1, as lower energy indicates that ligands and proteins form a complex with a higher binding affinity and greater stability. The docking interaction diagram of the optimal active site of the enzyme is shown in Figure 8A1,A2, and in 2D and 3D docking views, the interaction of MMP-1 amino acid residues with PMPPs is revealed (Figure 8).

Figure 8A3 show the interactions between PMPPs and the conventional hydrogen bonds, pi-alky and pi-sulfur bonds formed by PMPPs with amino acid residues His 228 were found. Van der Waals forces were also observed between AAPs and amino acid residues: Gln 186, Thr 230, Ser 229, Ser 227, Asn 160, Ser 172, Asn 171, Pro 173, and His 183. In addition, the alky was also observed between Pro 238 and PMPPs.

The optimal binding model of PMPPs (FAFQAAEIAQLMS)-EGFR1/FGFR1 is shown in Figure S3 with minimum binding energies of 8.76 kcal/mol and 7.93 kcal/mol, respectively (the lower the energy, the easier the pep-tide binds to the receptor protein). Therefore, it can be concluded that hydrogen bonding, Van der Waals forces, and covalent bond interactions are the primary interaction forces involved in the binding of PMPPs and MMP-1. These findings support and further confirm the results of tests conducted in vivo and in vitro.

## 3. Discussion

Wound repair is a complex and distinct process involving multiple cells and cytokines. The skin as a human body barrier is damaged for a series of complications such as ulcers, infections, scars, and even chronic wounds [18]. Clearly, this has prompted the need to develop drugs or products that meet the functional requirements of each stage of the healing process. At the same time, our results showed that topical and oral PMPs could both promote wound healing and inhibit scar formation, which was closely related to shortening the time of hemostasis and epithelialization, promoting angiogenesis, regulating cytokines, depositing collagen, and remodeling collagen fiber [15,16]. Hence, we purified the PMPs by gel chromatography, their biological activities were demonstrated by in vitro cellular assays, and the best fraction PMPs-4 (PMPPs) was screened by this method (Figure 1). This is closely related to the peptide sequence composition of PMPPs (Table 1), which is beneficial for promoting cell proliferation and laying a solid foundation for subsequent studies.

Molecular docking is an effective and reliable computational technique for predicting possible binding modes and studying the ligand binding mechanism between small molecules and proteins. Molecular docking is widely used in structural molecular biology and drug discovery [19], which is a useful technology to identify the binding mode or force of ligand protein complexes. Binding energy is also an important criterion for considering the interaction between proteins and ligands, and the lowest binding energy is considered to be more stable. Epidermal growth factor receptor (EGFR1), fibroblast growth factor (FGFR1), and matrix metalloproteinase (MMP-1) play important roles in cell proliferation, tissue remodeling, and wound healing [20]. Thus, it not only helped us to screen for the best peptide sequence FAFQAEIAQLMS, but also further elucidated the mechanism of the significant cell proliferation promotion by PMPPs.

Wound healing includes three phases that are intertwined in space and time, namely the inflammatory phase, the proliferative phase, and the tissue remodeling phase [21,22]. During the inflammatory period, macrophages secrete pro-inflammatory factors (IL-1β, TNF-α, IL-6, etc.) and anti-inflammatory factors (IL-10) to clear the necrotic tissue and prevent infection at the wound site [23,24]. On the third day after the trauma, macrophages in the skin tissue entered the injured area. Besides phagocytosing pathogens, they also secreted various growth factors and cytokines, as well as neutrophils [25]. Furthermore, the H&E microscopy showed that compared with the two control groups, the PMPPs group significantly inhibited the inflammatory response (Figure 5), which was closely related to the significant promotion of IL-10 secretion in the PMPPs group (Figure 4A,B).

The greatest feature of the proliferative period is the granulation tissue formed by fibroblasts, endothelial cells, and keratinocytes [26]. Meanwhile, at this stage, macrophages induce fibroblast proliferation by secreting TGF-β, and then some skin tissues produce collagen under the stimulation of TGF-β and regulate tissue repair by depositing the type III collagen [27,28]. The TGF-β/Smad signaling pathway plays an important role in cell growth, differentiation, migration, apoptosis, and repair after injury [29]. In addition, fibroblast growth factor-2 (FGF-2) and cyclin 1 (CCND1) play important roles in cell proliferation and tissue regeneration [30]. In this study, coated administration of PMPPs was effective in accelerating the process of wound epithelialization (Figure 5), and this was closely related to its significant promotion of the secretion of growth factors TGF-β, FGF-2, and CCND1, thereby favoring fibroblasts, epidermopoietic cells, and angiogenesis (Figure 4 and Figure 6). Importantly, the topical administration of PMPPs showed great potential to meet the functional requirements of all phases of wound healing, which are verified with the results of tissue remodeling (Figure 8 and Figure S3).

During the tissue remodeling period, macrophages secrete metalloproteinases, degrade excess fibers in the wound, induce apoptosis, and clear fibroblasts [31]. Meanwhile, collagen III levels increased significantly after the inflammatory phase, which can reduce scar formation. Type III collagen in the connective tissue is replaced by type I collagen in the remodeling period [32,33]. This was closely related to the fact that the PMPPs group significantly promoted the proliferation of CD31 and FGF (Figure 6), and enhanced collagen synthesis through the TGF-β/Smad signaling pathway (Figure S2). In addition, by shortening the inflammatory period, the PMPPs significantly promoted the secretion of TGF-β and cleverly coordinated the proportion of collagen in the wound, thereby inhibiting scar formation in PMPPs-treated mice, as evidenced through the results summarized in Figure 3 and Figure 7 [34]. Although positive drugs could promote skin wound healing and inhibit scar residue, the mechanism of action was different from PMPPs, which is consistent with the main efficacy reported: it was suitable for mild wound healing, and had the functions of promoting blood circulation, detoxification, pain relief, etc.

## 4. Materials and Methods

### 4.1. Materials

*Pinctada martensii* mantle were purchased from Dongfeng Market, Zhanjiang City, China. Neutral protease (3 × 10^4^ U/g) was purchased from Pangbo Biological Engineering Co., Ltd. (Nanning, China). Jing Wanhong ointment (Chinese medicine certificate Z20023137) was provided by Tianjin Da Rentang Jing Wanhong Pharmaceutical Co., Ltd. ELISA kits (Nanjing Jiancheng Biotechnology Research Institute); HaCaT and HSF cells were purchased from Beijing Beina Chuanglian Biotechnology Research Institute. IL-1β (interleukin-1β), TGF-β (transforming growth factor β1), TβRII (transforming growth factor beta 1 type II receptor), and Smad 7 (signal transduction molecule 7) were purchased from Mlbio, Shanghai, China. IL-10 (interleukin-10) was purchased from Nanjing Institute of Bioengineering, Nanjing, China. Human FGF-basic, purchased from Pepro Teck Company, USA. rhEGF was purchased from Meilun Biotechnology Co., Ltd., Dalian, China. Iodoacetamide, dithiothreitol, ammonium bicarbonate, and formic acid were purchased from Sigma Company, USA. Acetonitrile (>99.9%) was purchased from Fisher Chemical Company, USA. Fetal bovine serum, DMEM medium, PBS, 2.5%. Trypsin-EDTA, and penicillin-streptomycin double antibody were purchased from Thermo Fisher Scientific, USA. Sephadex G-25 was purchased from GE, USA. RPMI 1640 medium was purchased from Gibco, USA. BCA protein concentration determination kit (enhanced) was purchased from Shanghai Biyuntian Biotechnology Co., Ltd., China. The other chemicals used in this experiment were of analytical grade and are commercially available.

### 4.2. Preparation of PMPs

The preparation methods of PMPs were as reported by Yang et al. [16]: the *Pinctada martensii* mantle were washed, drained, and ground before being dissolved in water with a ratio of 1:3 (mantle: water). The hydrolysis reaction was carried out using neutral protease at 1000 U/g (raw material) at 53 °C, pH 7 for 3–5 h while stirring was carried out. Next, the mixture was incubated in boiled water for 10 min and cooled immediately to inactivate the enzymes. Following inactivation, the hydrolysate was fractioned into <3 K Da group using ultrafiltration system (XX42PMINI, Millipore, Burlington, MA, USA) and the ultrafiltration membranes (Mili Pellicon, Millipore, Burlington, MA, USA) and CNPs were obtained by rotary evaporation (N-1300V, EYELA, Tokyo, Japan) and freeze-drying (FDU-2110, EYELA, Tokyo, Japan) in sequence.

### 4.3. Gel Permeation or Ultrafiltration Purification of PMPPs

A suitable amount of Sephadex G-25 dry gum (GE, Boston, MA, USA) was soaked in 20% ethanol for 24 h to activate it. A 65 cm × 2.6 cm (long × inner diameter) gel column was filled it by the AKTA Purifier protein purification instrument (AKTA Purifier, Thermo, Waltham, MA, USA). Ultrapure water as the eluent was used to balance the pressure of gel column for 24 h when the gel column pressure was stable. After the ultrafiltration component of PMPs was passed through 0.22 μm membrane, the elution was 5 mL at a fixed flow rate of 1.0 mg·mL^−1^, and detected at 280 nm to obtain the small molecular purified peptides (PMPPs). The collected components were lyophilized for cell proliferation test.

### 4.4. Cell Proliferation Assay In Vitro

HaCaT (Human immortalized keratinocytes) and HSF (Human Skin fibroblast) cells were cultured in 25 cm^2^ cell culture flasks. Complete medium consisted of 90% DMEM, 10% fetal bovine serum, and 1% double antibody. The cultured HaCaT and HSF cells were diluted into 2 × 10^5^ cells·mL^−1^ and 100 μL per well was added into 96-well microplates and cultured at 5% CO_2_ and 37 °C saturated humidity for 4 h. After the cells were completely adherent, the PBS was used to gradient formulation of SMPs and purified components of different concentrations. After passing the samples through 0.22 μm filter membrane, 10 μL of each was added to the adherent cells. WST-8 (tetrazolium salt 2-(2-methoxy-4-nitrophenyl)-3-(4-nitrophenyl)-5-(2,4-disulfophenyl) -2H-tetrazolium, monosodium salt (CCK-8)) diluted with 100 μL of culture medium was added after 24 h of culture for reaction for 1 h and then OD value was measured at 450 nm.

### 4.5. Peptide Sequence Analysis of PMPPs

The major peptide sequence analysis of PMPPs was as reported by Yang et al. [16]: The PMPPs lyophilized powder were reduced by 10 mM DTT for 1 h at 56 °C, then alkylated by 50 mM IAA in dark for 40 min at room temperature. Following the addition of the enzyme, the solution was incubated overnight at 37 °C. Salt from the peptide solution was removed using a C18 tip, and peptides were then lyophilized to near dryness. Finally, Peptide sequences were identified by electrospray ionization mass spectrometry and tandem mass spectrometry (ESI-MS/MS) in positive ion mode. After chromatography, ESI-MS/MS was carried out using a Q Exactive™ triple quadrupole instrument (Thermo Fisher Scientific, Waltham, MA, USA) equipped with an ESI source. Sequences of characteristic peptides were determined by analysis and comparison with secondary fragments of peptides from the collision-induced dissociation spectrum of the protonated molecule [M + H] + in the Uniprot database.

### 4.6. Animal Grouping and Establishment of Trauma Model

The study was approved by the Guangdong Ocean University (Zhanjiang, China), Experimental Animal Care Ethics Committee (Approval No.: 20190001, Approval Date: 17 June 2019). SPF male mice (4-weeks-old) were purchased from Pengyue Experimental Animal Breeding Co., Ltd. (Jinan, China) and the production license number was SCXK 20190003, Shandong, China. The laboratory license number was SYXK 2019-0204, Guangdong, China. All experiments were in accordance with the ARRIVE guidelines and were conducted in accordance with the National Institutes of Health guidelines for the care and use of laboratory animals (NIH Publication 8023, revised 1978).

The dorsal hair was shaved and sterilized before the experiment, and 1% pentobarbital was administered as sodium (50 mg/kg) to anesthetize the mice. A full skin defect model of 8 mm in diameter was made on the back of each SPF grade KM male mice (20.0 ± 2.0 g, 4 weeks old). Fifty-seven model mice were randomly divided into three groups: negative control group, positive control group (Jing Wanhong ointment was selected as the positive control drug, which has the effects of activating blood circulation, detoxifying, detumescence and pain relieving, and removing saprophytic muscles. The active ingredients are mainly Ampelopsis radix, Angelica dahurica and other traditional Chinese medicines; dose: 2–3 mg/day, administration method: Topical use), and PMPPs group (Topical use, peptides dose: 0.5–1 mg/day), and 19 rats were in each group. The negative control group was not administered after modeling, and the positive control group and the PMPPs group were administered on the day of modeling, and were applied once a day for 18 days.

### 4.7. Percentage of Wound Closure and Residual Scar Rate

Photographs were taken at a fixed height every two days, and the image processing software Image J (National Institutes of Health, Bethesda, MD, USA) was used to analyze and calculate the wound healing rate and observe the scab removal time and wound scar formation [15].

### 4.8. Tissue Preparation for Histological Assessment

Tissues were removed around each mouse wound, immersed in neutral formalin, and then dehydrated with a series of increasing concentrations of alcohol. Dehydrated tissue was embedded in paraffin to prepare sample sections for histopathological and histomorphological observation [16].

### 4.9. ELISA Analysis

After centrifugation at 10,000 rpm for 15 min at 4 °C, the supernatant of the homogenized tissue was collected as protein extract. Before the index detection, the protein content of each sample (Beyotime Biotechnology Co., Ltd., Shanghai, China) was measured and diluted to the same level. Quantification of IL-1β, TGF-β1, TβRII, Smad7, and IL-10 in 10% (*v*/*v*) dorsal skin homogenate supernatant was performed using ELISA kits (Nanjing Jiancheng, Nanjing, China) [15].

### 4.10. Hematoxylin and Eosin Staining for Microscopic Analysis

The tissue was taken from the injured part of the back of the mouse, and the tissue was immediately fixed in formalin. After dehydration with ethanol solution, the sections were stained with hematoxylin and eosin. In order to observe the histopathological changes, the sections were examined at a magnification of 40 times using Olympus microscope (Olympus IX51, Tokyo, Japan).

### 4.11. Immunohistochemistry

After dehydration, hydration, and hydrothermal antigen repair, the antigen was extracted with citrate buffer (pH 6.0). Goat serum was added to block the non-specific binding site and incubated at room temperature for 20 min. Next, 50–100 microliters of primary antibody (diluted 1:200, provided by Nanjing Institute of bioengineering, China) were added to the tissue sections drop by drop, and the slides were placed in a humidification chamber at 37 °C for 2 h. Then, 50 μL universal second-class IgG antibody Fab fragment HRP polymer (provided by Nanjing Institute of bioengineering, China), was added, followed by incubation at 37 °C for 30 min, washing with PBS for 3 times. After adding diaminobiphenylamine (DAB) to make it colored, CD31, EGF, and FGF were observed under optical microscope (Olympus IX51, Tokyo, Japan).

### 4.12. Sirius Red Picric Acid Dyeing

The steps of the Sirius red staining method were as follows [15]: Firstly, the sample was fixed in 10% formalin fixative and routinely dehydrated. Second, sample sections were stained with Sirius Red staining solution for 8–10 min. Subsequently, the running water was quickly rinsed to remove the stained surface of the section. Finally, the anhydrous ethanol was quickly dehydrated to transparency and sealed with a neutral gum.

### 4.13. Molecular Docking

The crystal structure of protein receptors linked to skin wound healing of EGFR1 (PDB code: 3POZ, resolution: 1.50 Å), FGFR1 (PDB code: 1AGW, resolution: 2.40 Å) [35] and MMP-1 (PDB code: 966C, resolution: 1.90 Å) [36] was obtained from RCSB Protein Data Bank (https://www.rcsb.org/, access on 13 September 2021). The initially crystal structure of EGFR1 was processed with AutoDock Tools 1.5.6 by removing non-polar water molecules, adding polar hydrogen, and saving the original charge of EGFR1/ FGFR1/ MMP-1 before exporting as a .pdbqt file, which was processed with AutoDock Tools 1.5.6 to form the .pdbqt file for the docking study. In the docking process, the receptor was kept rigid while making the ligands more flexibly during simulation.

The probable interaction between SMPPs (FAFQAEIAQLMS) and EGFR1/ FGFR1/ MMP-1 were explored through Autodock 4.2 with the grid box centered at the coordinates of x = 126, y = 126, and z = 126. The grid sizes along the X, Y, and Z axes were set to 60 × 60 × 60 at a grid space of 0.375 Å (since the specific binding site of the peptide to the protein receptor was unknown, to avoid measures of optimal binding sites, a full domain boxing of the receptor was chosen for semi-flexible docking with the peptide). The docking calculations were performed 100 times by Lamarckian genetic algorithm, and the output was sorted by Lamarckian GA module. The protein–ligand complexation possesses the minimum energy scoring was selected for further visualization of the docked conformation with PyMOL and Discovery Studio.

### 4.14. Data Analysis

The wound area was calculated with Image J software (National Institutes of Health, Bethesda, Maryland, USA), and the experimental data were expressed as means ± standard deviation (mean ± S.D.). SPSS20 software (IBM, Armonk, NY, USA) was used for statistical analysis, and the LSD method was used for multiple comparisons between groups; p-values of less than 0.05 were considered to be statistically significant.

## 5. Conclusions

Studies showed that the PMPPs screened by gel chromatography purification and cellular assays were composed of 33 peptides that could achieve effective scarless healing though promoting the inhibition of inflammatory response and cytokine secretion, accelerating the epithelialization process, and activating the TGF-β/Smad pathway to coordinate the trauma type I/III collagen ratio. The molecular docking technique not only helped us to screen for the most promising peptide sequence FAFQAEIAQLMS, but also elucidated that FAFQAEIAQLMS played a role in promoting wound healing by docking easily with the protein receptor EGFR1/FGFR1/MPP-1, which enriches the pro-healing mechanism of PMPPs. This research aimed to provide new materials and new methods for the development of wound healing drugs.

## Figures and Tables

**Table 1:** Main peptide sequences analysis of PMPPs.

Sequence	Peptide Sequence of PMPPs	Molecular Mass (Da)	Score
1	RGVVDSEDLPLNISRE	1512.78	58.46
2	KEAFSLFDKDGDGTITTKE	1843.89	56.77
3	FIMDNCEELIPEYLN	1653.73	47.74
4	RYESLTDPSKLDSGKD	1538.75	46.45
5	RELISNSSDALDKIRY	1559.82	45.27
6	FAFQAEIAQLMS	1136.56	42.91
7	RELISNSSDALDKI	1290.63	41.91
8	FAFQAEIAQLMS	1120.56	41.40
9	KFYEQFSKN	947.43	39.76
10	KHFSVEGQLEFRA	1347.66	39.38
11	LISNSSDALDKIRYE	1480.75	38.97
12	KLTDEEVDEMIRE	1348.62	38.78
13	FIMDNCEELIPEYLN	1637.73	36.49
14	KYRHPDGSYSA	1080.46	36.41
15	FLRELISNSSDALDKIRYE	1992.06	33.79
16	YSNKEIFLRELI	1247.69	33.05
17	KLTDEEVDEMIRE	1364.62	32.39
18	YESLTDPSKLD	988.51	31.40
19	RHVMTNLGEKL	1043.51	30.61
20	WEDHLAVKHFS	1094.55	30.55

Note: Scores obtained by scoring a known protein database to measure the similarity between theoretical mass spectra and experimental mass spectra. Peptide sequences scoring ≥30 or more were considered to be present with higher confidence.

## Data Availability

Not applicable.

## Supplementary Materials

The following supporting information can be downloaded at: https://www.mdpi.com/article/10.3390/md20070417/s1, Figure S1: H&E stain histological analysis (40×); Figure S2: Effects of topical administration of PMPPs on TGF-β/Smad signaling pathway. Figure S3: Docking results of PMPPs (FAFQAEIAQLMS) with protein receptors EGFR1/FGFR1.
