# Supplementary material for: Investigation of the In Vivo, In Vitro, and In Silico Wound Healing Potential of Pinctada martensii Purified Peptides
Source: Mar Drugs. 2022 Jun 26;20(7):417. doi: 10.3390/md20070417 (PMC9325270; doi:10.3390/md20070417)
Supplement: Supplementary file 1 [file marinedrugs-20-00417-s001.zip › marinedrugs-1733167-supplementary.pdf]

Investigation of the *in vivo*, *in vitro* and *in silico* wound healing potential of *Pinctada martensii* purified peptides

Ting Zhang[1, †], Faming Yang[1,2, †], Xiaoming Qin[1,3,4,5,6,7,*], Xianmei Yang[1], Chaohua Zhang[1,3,4,5,6,7], Zhaoyi Wan[1] and Haisheng Lin[1,3,4,5,6,7]

[1] College of Food Science and Technology, Guangdong Ocean University, Zhanjiang 524088, China; ZhTing95@163.com (T.Z.); yangfm0123@163.com (F.Y.); 13414884976@163.com (X.Y.); Zhangch2@139.com (C.Z.); spring5water@163.com (Z.W.); haishenglin@163.com (H.L.)

[2] Marine College, Shandong University, Weihai, 264209, China

[3] Guangdong Provincial Key Laboratory of Aquatic Product Processing and Safety, Zhanjiang 524088, China

[4] National Research and Development Branch Center for Shellfish Processing (Zhanjiang), Zhanjiang 524088, China

[5] Guangdong Province Engineering Laboratory for Marine Biological Products, Zhanjiang 524088, China

[6] Guangdong Provincial Engineering Technology Research Center of Marine Food, Zhanjiang 524088, China

[7] Collaborative Innovation Center of Seafood Deep Processing, Dalian Polytechnic University, Dalian 116034, China

* Correspondence: qinxm@gdou.edu.cn; Tel.: +86-0759-2396027 (M.Q.)

† These authors contributed equally to this work.

Corresponding Author

*****XiaoMing Qin, Ph.D.

College of Food Science and Technology, Guangdong Ocean University, No.1, Haida Road, Zhanjiang 524000, China

Tel: +86 0759 2396027

Email: xiaoming0502@21cn.com

Supplementary Material and Methods

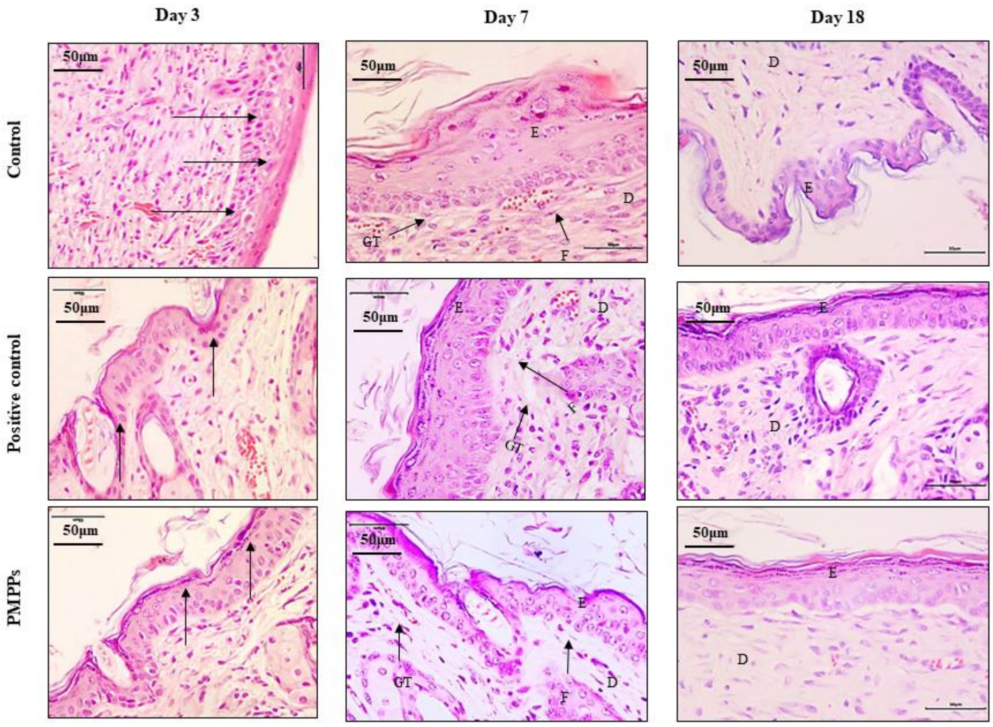

Figure S1. H&E stain histological analysis (40×). Note: Black thick arrows indicate inflammatory cell infiltration. Letters D, E, F, and GT represent the dermis layer, the epidermal layer, fibroblasts, and granulation tissue, respectively.

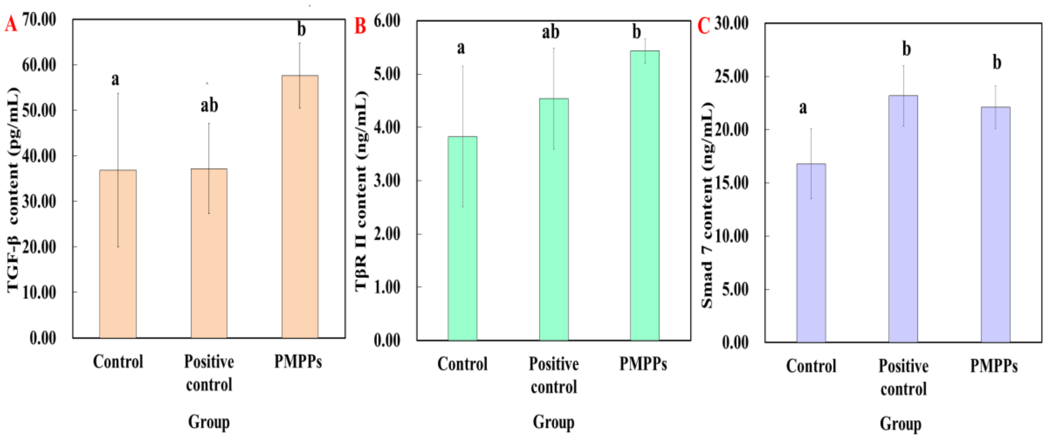

Figure S2. Effects of topical administration of PMPPs on TGF-β/Smad signaling pathway. (A) Effect of PMPPs on the expression of TGF-β. (B) Effect of PMPPs on TβRII expression. (C) Effect of PMPPs on Smad 7 expression. Note: Different superscript letters on the same day indicate significant ($p < 0.05$) and non-significant ($p > 0.05$) differences between groups, respectively.

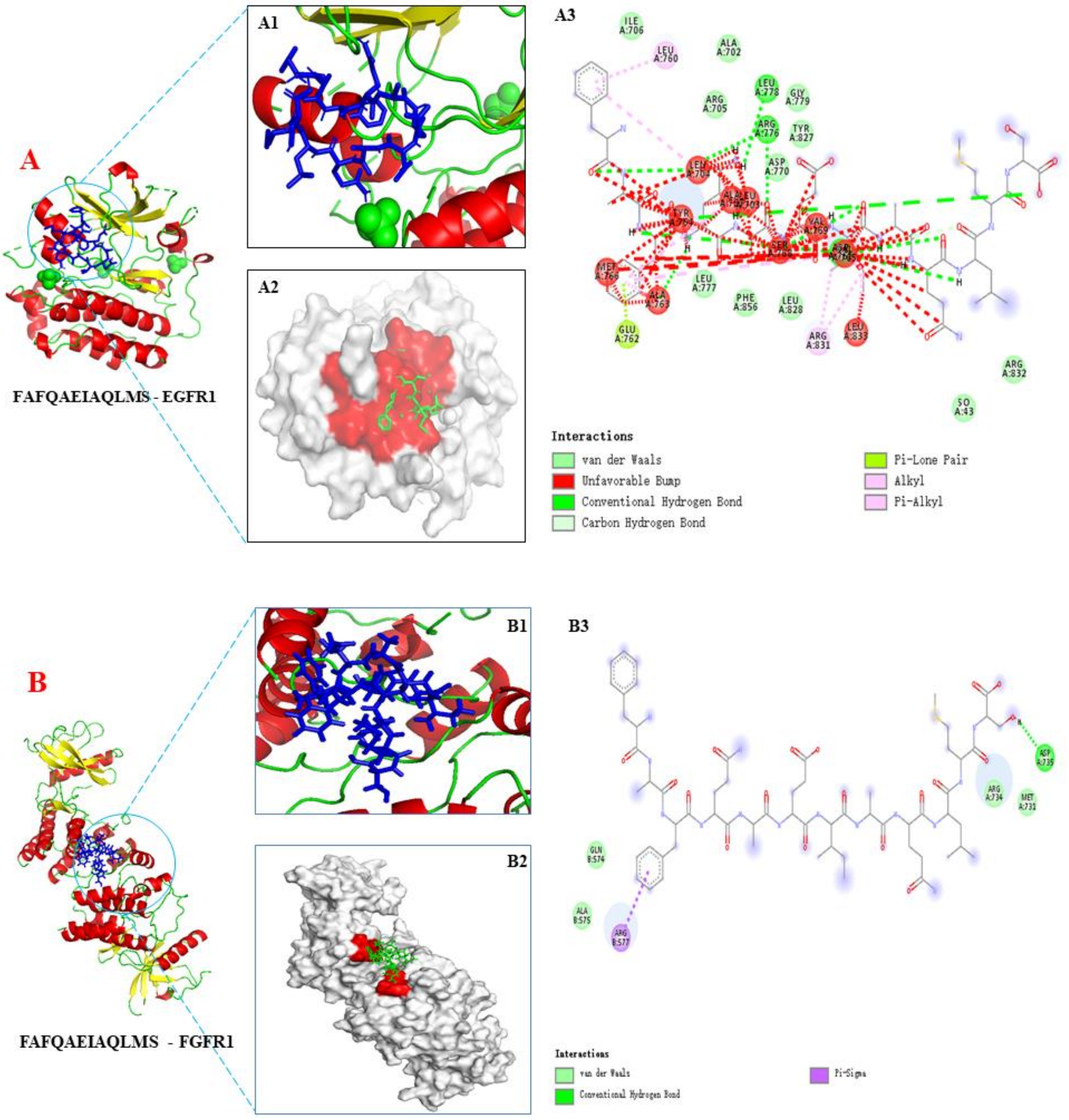

Figure S3. Figure 8. The docking results of PMPPs (FAFQAEIAQLMS) with protein receptors EGFR1/FGFR1. (A1-2 and B1-2) 3D structure of PMPPs - EGFR1/FGFR1 and the process of binding interaction between them. (A3 and B3) 2D interaction diagram of PMPPs - EGFR1/FGFR1.
